# Evolutionary Insights Into Microbiota Transplantation in Inflammatory Bowel Disease

**DOI:** 10.3389/fcimb.2022.916543

**Published:** 2022-06-22

**Authors:** Xiaoli Wang, Jingwen Zhao, Yuanhang Feng, Zelin Feng, Yulin Ye, Limin Liu, Guangbo Kang, Xiaocang Cao

**Affiliations:** ^1^ Department of Gastroenterology and Hepatology, Tianjin Medical University General Hospital, Tianjin Institute of Digestive Disease, Tianjin Key Laboratory of Digestive Diseases, Tianjin, China; ^2^ Department of Biochemical Engineering, School of Chemical Engineering and Technology, Tianjin University, Tianjin, China; ^3^ Frontiers Science Center for Synthetic Biology and Key Laboratory of Systems Bioengineering (Ministry of Education), Tianjin University, Tianjin, China; ^4^ Institute of Shaoxing, Tianjin University, Zhejiang, China

**Keywords:** microbiota transplantation, artificial consortium transplantation, combination principles, clinical study, inflammatory bowel disease

## Abstract

The intestinal microbiome plays an essential role in human health and disease status. So far, microbiota transplantation is considered a potential therapeutic approach for treating some chronic diseases, including inflammatory bowel disease (IBD). The diversity of gut microbiota is critical for maintaining resilience, and therefore, transplantation with numerous genetically diverse gut microbiota with metabolic flexibility and functional redundancy can effectively improve gut health than a single probiotic strain supplement. Studies have shown that natural fecal microbiota transplantation or washing microbiota transplantation can alleviate colitis and improve intestinal dysbiosis in IBD patients. However, unexpected adverse reactions caused by the complex and unclear composition of the flora limit its wider application. The evolving strain isolation technology and modifiable pre-existing strains are driving the development of microbiota transplantation. This review summarized the updating clinical and preclinical data of IBD treatments from fecal microbiota transplantation to washing microbiota transplantation, and then to artificial consortium transplantation. In addition, the factors considered for strain combination were reviewed. Furthermore, four types of artificial consortium transplant products were collected to analyze their combination and possible compatibility principles. The perspective on individualized microbiota transplantation was also discussed ultimately.

## 1 Introduction

Inflammatory bowel disease (IBD) is an inflammatory disease of the intestine, including ulcerative colitis (UC) and Crohn’s disease (CD). The disease has complex etiology, but it is generally related to genetics, immune response, environmental factors and the gut microbiome (West et al., 2017). The current IBD treatment relies on anti-inflammatory agents, immunosuppressants, and biologic agents (Van Assche et al., 2013; Gomollón et al., 2016). Even so, these agents do not achieve satisfactory outcomes in some patients, underlining the need for alternatives. Microbiome dysbiosis is an essential feature of IBD (Maloy and Powrie, 2011), which makes regulating the gut microbiome as one of the potential strategies for IBD treatment.

The human gut is home to numerous microbiota, which form a complex gut microbiome community. The gut microbiome is a highly dynamic and intricate ecosystem that differs among individuals, influenced by host genetics, age, diet, drug use, and other factors (Costello et al., 2009; Yatsunenko et al., 2012; Duffy et al., 2015; Xie et al., 2016). In a healthy state, maintaining a dynamic balance of gut microbes exists in healthy individuals. Disrupting this balance can cause several human diseases such as metabolic syndrome (Lim et al., 2017), obesity (Liu et al., 2017), infections (Petrof et al., 2013), gastrointestinal diseases [irritable bowel syndrome (IBS) (Pimentel and Lembo, 2020) and IBD (Chu et al., 2016)]. Introducing microorganisms into the intestinal tract can rapidly reverse diseases related to gut microbial diversity and abundance imbalance. Replacing the missing symbiotic microbes in the gut with corresponding strains or a mix of specific strains may prevent or treat such conditions. The gut microbiome includes bacteria, fungi, archaea and viruses. Recently, Underhill et al. reviewed the important role of fungal microbiome regulation in the development and severity of IBD in some patients (Underhill and Braun, 2022). The bacterial microbiome accounts for a large proportion of the intestinal tract which plays a major role in intestinal disturbance. Therefore, in this review, we only focused on the treatment of IBD through bacterial microbiome. Gut microbe-based therapies such as fecal microbiota transplantation (FMT) (Paramsothy et al., 2017), washed microbiota transplantation (WMT) (Zhang et al., 2020), and live biotherapeutic products (LBP) (Ye et al., 2021)have been used to treat IBD related to microbial alteration.

The essential characteristics of the gut microbiome are stability and resilience (Lozupone et al., 2012). Without interference, the gut microbiome remains stable. The gut microbiota is generally highly resilient to disturbances, and thus, the abundance of numerous key species remains stable in the host for a period of time. The stability and resilience of gut microbiota are closely related to their diversity. Higher microbial diversity increases the functional redundancy levels. It is generally thought to play a critical role in stabilizing microbial community function during disturbances (Fassarella et al., 2021). Therefore, transplantation with the combination of multiple microorganisms is more effective in modulating gut health than with a single probiotic strain supplement.

Studies have shown that fecal microbiota transplantation can alleviate IBD (Moayyedi et al., 2015; Sokol et al., 2020). However, FMT also causes adverse reactions in some patients due to the complex components in transplants (Wang et al., 2016; DeFilipp et al., 2019). A washed microbiota for transplantation that minimizes the adverse reactions caused by natural FMT has been developed (Zhang et al., 2020). Nevertheless, the precise composition of the transplantation flora is unclear, and the procedure has potential health risks. The recent technology has deepened our understanding of microbial community and its application for IBD therapy. FMT can be performed through enema, orally through freeze-dried bacterial capsules (Crothers et al., 2021), or non-freeze-dried bacterial suspension capsules (Khanna et al., 2021). Beneficial bacteria can be isolated from fermented foods or feces or engineered to obtain desirable biological characteristics (Kurtz et al., 2019; Puurunen et al., 2021).

The influence factors of microbiota transplantation outcome include recipient factors and transplant factors. The recipient parameters, such as genetics, immunity, microbiota and lifestyle, affect the efficacy of microbiota engraftment (Danne et al., 2021). Moreover, the matching between donors and recipients is essential for the long-term maintenance of disease remission (Wilson et al., 2019; Okahara et al., 2020). Given the limitation of the length of the article, we only discussed the diverse microbiota transplantation for IBD treatment.

To sum it up, numerous gut microbiota is vital to human health. Numerous studies have confirmed that intestinal flora participates in intestinal maturation and homeostasis through multiple functions, while symbiosis and interacting with human cells and organs (Backhed et al., 2005; Human Microbiome Project Consortium, 2012). Gut microbiota therapy can be performed using FMT and WMT, and this procedure effectively alleviates microbiome-related disorders, including IBD. However, those are not the best choices due to undefined composition. Microbial therapy with clear composition and standard quality monitoring may be the direction of microbiota transplantation. This review focuses on the current research on different artificial consortium transplant products and their improvement for IBD treatment. We discussed the characteristics of the strains used for combination and their possible compatibility principles, which may be necessary for the better development of individualized microbiota transplantation.

## 2 Undefined Consortium Transplantation

This refers to a community of microbiome with unknown composition.

### 2.1 Natural Microbiota Transplantation

Natural microbiota transplantation, also known as fecal microbiota transplantation (FMT), is a procedure in which stool from a healthy donor is delivered to the intestines of a recipient patient through enema or oral capsules (Gupta and Khanna, 2017). FMT is currently used primarily to treat recurrent *Clostridioides difficile* infections (Hvas et al., 2019). However, even though the mechanism of action of FMT is not well understood, existing findings show that it generally restores the abnormal composition and abundance of the gut microbiota. FMT is effective against microecological disorders, including IBD, hepatic encephalopathy (HE), metabolic syndrome, IBS, autism, and cancer (Bajaj et al., 2017; de Groot et al., 2017; Kang et al., 2017; Fong et al., 2020; Skrzydło-Radomańska et al., 2021).

#### 2.1.1 Clinical Studies of FMT in IBD

Using a randomized controlled trial, Moayyedi et al. revealed that FMT induced remission of active UC. FMT is even more effective against early UC because it is easier to restore the early gut microbial imbalance (Moayyedi et al., 2015). Another study confirmed that a 2-donor fecal microbiota preparation (FMP) was also a safe and effective method for restoring the normal intestinal microbial diversity in patients with active UC (Jacob et al., 2017). In addition to direct colon FMT, oral capsule FMT (cFMT) is well tolerated in mild to moderate UC patients (Crothers et al., 2021). Also, there are currently several registered trials investigating the efficacy of cFMT in IBD, as the previous Halaweish et al. described (Halaweish et al., 2022). Oral cFMT is a more acceptable alternative for UC treatment than the direct colon FMT, and it may enhance the potential of long-term microbial-based treatment strategies. Sokol et al. conducted a pilot randomized controlled study showed that FMT significant decreased endoscopic activity and C relative protein level of CD patients (Sokol et al., 2020). A systematic review evaluated the efficacy of FMT in Crohn’s disease, involved 13 cohort studies and two RCTs between 2014 and 2020, showed that FMT may be an effective and safe therapy for CD and needed large controlled trials to confirm (Fehily et al., 2021).

The efficacy and safety of FMT have been studied in adult with IBD and children with UC and CD. Nikhil Pai et al. conducted a 6-week randomized, placebo-controlled pilot study using FMT in children with UC and CD to evaluate the safety and effectiveness of FMT supplement, providing preliminary evidence for the clinical application of FMT in children with IBD (Pai and Popov, 2017; Pai et al., 2019). Katarzyna et al. further demonstrated that FMT is also a safe and effective alternative for treating cytomegalovirus colitis in children with UC (Karolewska-Bochenek et al., 2021).

#### 2.1.2 The Limitations of FMT

Even though FMT is effective against IBD remission, its effectiveness cannot be controlled by humans and is related to the diversity of the fecal microbiota in the donor individual, and there are no reliable and stable sources of the feces. The donor fecal microbiota mixture has many unknown ingredients, including bacteria, yeasts, parasites and viruses. It is unclear which one is responsible for beneficial effects and which may pose a risk by transferring antibiotic resistance or producing genotoxic metabolites. In addition, given the inter- and intra-individual differences in gut microbiota, the transplantation effects are not uniform even with the same donor.

Due to the complex composition of FMT, some adverse reactions often occur. Mild to moderate adverse reactions include abdominal pain, flatulence, increased stool frequency, constipation, vomiting, belching, fever, whereas serious adverse effects include viral and bacterial infections, relapse of IBD, and death (Wang et al., 2016). In 2019, the Food and Drug Administration (FDA) reported two cases of serious adverse events of *Escherichia coli* bacteremia that produces extended-spectrum beta-lactamase (ESBL) after FMT. Genetic sequencing revealed that both patients received FMT from the same donor. one of the patients died (DeFilipp et al., 2019). Moreover, optimized screening of fecal bacteria transplantation donors did not seem to improve the efficacy of FMT in the treatment of active UC (*ECCO 2022 abstract OP03*). Although studies have shown the safety and efficacy of multi-donor FMT for diseases, including IBD and obesity (Jacob et al., 2017; Wilson et al., 2021), there is no clear method for selecting multiple donors, and there is no study directly comparing the efficacy of single donor and multi-donor FMT.

### 2.2 Processed Microbiota Transplantation

Processed microbiota transplantation, known as washed microbiota transplantation (WMT), is the microfiltration of feces to remove fecal solids, parasites, and fungi from feces suspension. Pro-inflammatory metabolites such as leukotriene B4, corticosterone and prostaglandin G2, are also removed from the feces. Regarding safety, quality control and precise bacteria enrichment, Zhang et al. first revealed that WMT is superior to FMT through clinical results, animal experiments and *in vitro* trials (Zhang et al., 2020). The incidence of WMT-related adverse events in CD patients (since April 2014) was 8.7%, significantly lower than 21.7% in patients with manual FMT (from 2012 to April 2014) (Wang et al., 2018).

#### 2.2.1 Clinical Studies of WMT in IBD

Based on a single-center, open-label prospective study, Chen et al. revealed that washed-treated FMT safely and effectively achieved a clinical response in 77.8% (7/9) of the assessed UC patients in just two weeks. At week 12, achieved clinical remission in 55.6% (5/9), whereas the endoscopic response rate was 33.3% (3/9) (Chen et al., 2020). A separate study showed that clinical remission was achieved in 53.7% of IBD patients after WMT therapy, and this therapy significantly increased the colonization rate of Akkermansia, a beneficial bacteria. Thus, the efficacy of WMT in treating IBD may be closely related to the abundance of Akkermansia bacteria (Zhang et al., 2020). Zhang and colleagues reported a case study of UC patients with recurrent fungal infections in which the antifungal therapy had failed. Interestingly, repeated WMT therapy remarkably and rapidly decreased the serum concentration of inflammatory makers and cleared the fungal during hospitalization. The fungal infection had not recurred after 6-month follow-up. However, the clinical application of WMT for recurrent fungal infections treatment needs further investigation (Wu et al., 2021). A randomized, open clinical study showed that enteral nutrition in combined with early WMT could rapidly improve the nutritional status and induce clinical remission in CD patients with malnutrition (Xiang et al., 2021).

## 3 Artificial Consortium Transplantation

We defined artificial consortium (AC) in a narrow sense as a combination of microbiome with clear composition in a specific manner. And the way AC are transplanted into the gut is called artificial consortium tranplantation (ACT).

### 3.1 Advances in IBD

#### 3.1.1 *In Vitro* Studies of ACT

Geirnaert et al. investigated the therapeutic potential of a mix of six butyric-producing bacteria against IBD, given the beneficial effects of butyric acid on epithelial barrier function and intestinal health. They found that the bacterial mix significantly enhanced the colonization of related butyric-producing bacteria and improved the integrity of the epithelial barrier *in vitro (* Geirnaert et al., 2017). Pistol et al. found that a combination of grape pomace (GP) extract and a mixture of Lactobacillus bacteria modulated inflammation by regulating the expression of related genes (Pistol et al., 2019). Palócz et al. reported the effect of chlorogenic acid in combination of *Lactobacillus plantarum* 2142 in reducing the lipopolysaccharide (LPS)-induced intestinal inflammation in porcine IPEC-J2 cells (Palócz et al., 2016).

Cuffaro et al. developed a method of evaluating the function of 21 strains isolated from neonatal and adult gut microbiota. They found that the isolated strains regulated the immune response and enhanced the functioning of the epithelial barrier. Also, 33% of the isolates exerted various benefits (Cuffaro et al., 2021). Even so, more *in vitro* studies are needed to identify the specific species, strains, or metabolites important for health, which extends the selection of a limited number of bacteria considered to have clinical importance and potential health-beneficial properties.

#### 3.1.2 Preclinical Studies of ACT

VSL#3 is the most studied probiotic combination used against IBD. Each VSL#3 dose contained 450 billion freeze-dried bacteria (*Streptococcus thermophilus, Bifidobacterium longum, B. breve, B. infantis, Lactobacillus acidophilus, L. plantarum, L. casei, L. bulgaricus*) and corn starch (Reiff et al., 2009). Current, VSL#3 is being used to IBD clinical treatment. Vivomixx^®^ in EU, Visbiome^®^ in USA, similar component to VSL#3, also alleviated canine colitis by increasing mucosal polyamine levels and TJP expression (White et al., 2017; Rossi et al., 2018). Biagioli et al. formulated a new probiotic combination by adding Bacillus subtilis to Vivomixx^®^. This formulation enhanced the beneficial effects of Vivomixx^®^ on DSS and TNBS-induced colitis (Biagioli et al., 2020).

Lactobacillus and Bifidobacterium are the most common probiotics. The efficacy of Lactobacillus in combination with Bifidobacterium against IBD, such as Ultrabiotique^®^ (*Lactobacillus acidophilus, Lactobacillus Plantarum, Bifidobacterium lactis and Bifidobacterium breve*), Citogenex (*L. casei, Bifidobacterium lactis*), PM2 (*Lactobacillus acidophilus, Lactobacillus paracasei, Lactobacillus rhamnosus, and Bifidobacterium lactis*) has been evaluated. Ultrabiotique^®^ alleviated intestinal inflammation and maintained the mucosal barrier in mice with DSS-induced colitis (Toumi et al., 2013). Pre-administration of Citogenex can alleviate TNBS-induced colon injury (Traina G, 2016). PM-2 attenuated 5-FU-induced mucositis, increasing villus/crypt ratio while decreasing inflammation in the intestine (Quaresma et al., 2020). Bacterial strains from different isolated sources can be combined. Je et al. evaluated the efficacy of *Lactobacillus johnsoniil* DCC9203 in combination with *Bifidobacterium animal subspecies lactis* IDCC4301 isolated from the feces of infants with *Lactobacillus plantarum* IDCC3501 isolated from the pickle at a ratio of 1:1:1 to form ID-JPL934, was applied to DSS-induced colitis model. It was found that ID-JPL934 could reduce mucosal and submucosal immune cell infiltration and decrease intestinal cell loss (Je et al., 2018).

In addition to the common Lactobacillus and Bifidobacterium combinations, some specific strain combinations have also been studied in animal models of IBD. Ming Li et al. demonstrated that a combination of 10 fecal bacteria, called bacterial consortia transplantation (BCT), was comparable to FMT in reestablishing mucosal barrier function in mice with intestinal disorders and that BCT was more stable and controllable than FMT (Li et al., 2015). Further investigation revealed that BCT rapidly restored intestinal microbiome balance, renovated the interaction between symbiotic flora and intestinal γδT17 cells, and improved mucosal barrier function (Li et al., 2016; Li et al., 2017). van der Lelie et al. further reported that gut-103, a cocktail of 17 bacterial strains, rapidly colonized mice intestines and alleviated experimental colitis established in germ-free mice. They further modified unsuitable bacterial strains, including antibiotic resistance, pathogenic, and strict anaerobes, to formulate a complex cocktail of 11 bacterial strains named GUT-108. This bacterial formulation induced stable and prolonged intestinal tract colonization, providing redundancy protection (van der Lelie et al., 2021). Clinical trials of similar probiotic preparation named I3.1 and comprising *Lactobacillus plantarum* (CECT7484, CECT7485) and *Pediococcus acidilactici* (CECT7483) alleviated IBS. Lorén et al. further demonstrated that I3.1 probiotic protected against DSS-induced colitis and IL-10-deficient colitis in mice (Lorén et al., 2017). GI7, composed of four lactobacillus, two Bifidobacterium species, and *Streptococcus thermophilus*, significantly inhibited the production of innate pro-inflammatory cytokines and relieved DSS-induced colitis (Kim et al., 2017). Commercial products Aviguard^®^, which comprise ten different bacterial species, alleviated acute enterocolitis induced by campylobacter bacteria (Heimesaat et al., 2021).

#### 3.1.3 Clinical Studies of ACT

**Figure 1:**
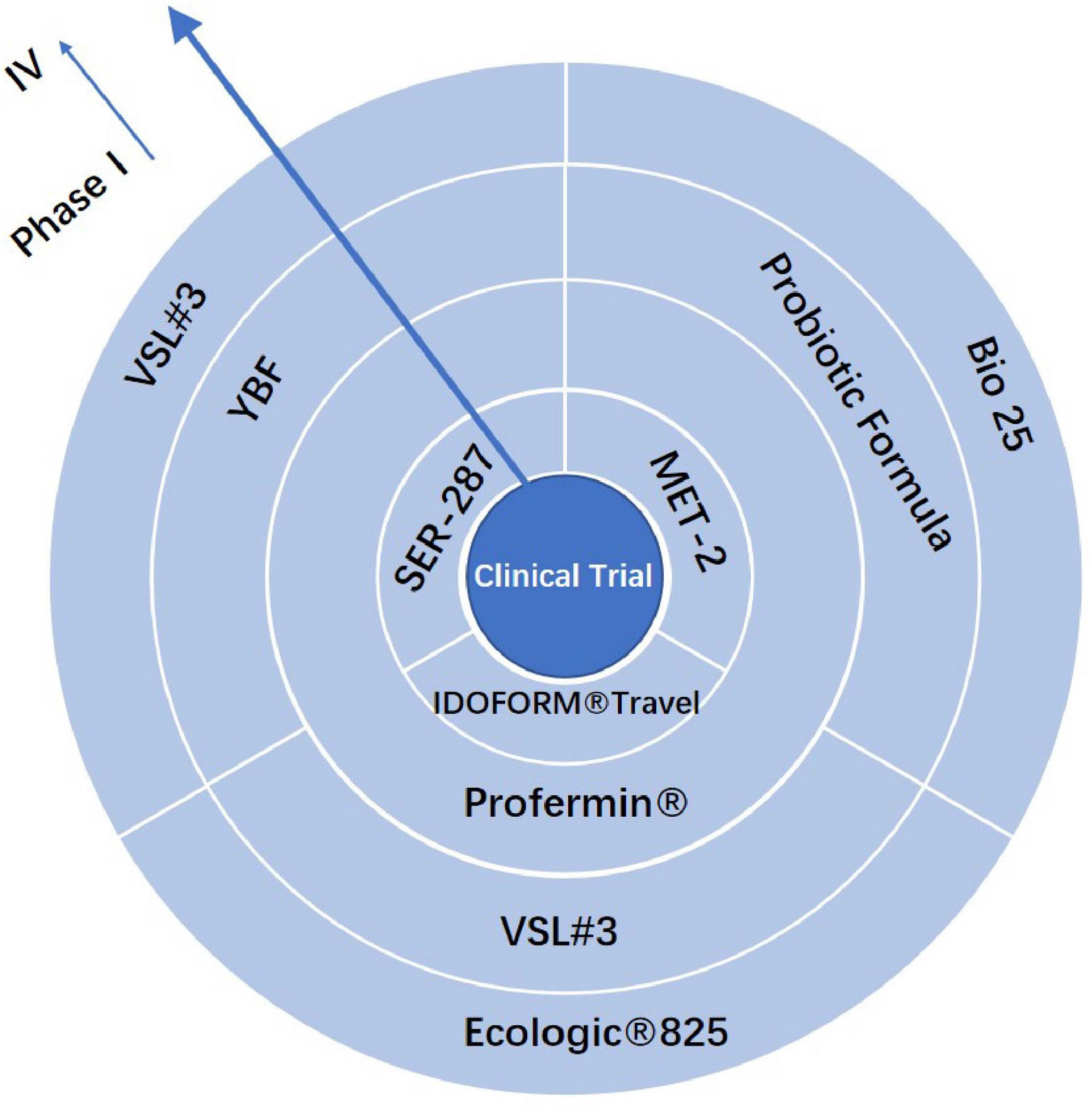
Artificial consortium transplantation products with clinical trials.

Clinical trials must be conducted to verify the safety and efficacy of treatment formulations, including bacterial combination therapy (**Figure 1**). Several studies have demonstrated the effect of VSL#3 on IBD in mice, rats, and dogs in last decade. VSL#3 prevented the apoptosis of intestinal epithelial cells, promoted the expression of the intestinal tight junction protein (TJP), reduced the production of pro-inflammatory factors, regulated the functioning of T cells and macrophages, and changed the composition of intestinal microorganisms (Reiff et al., 2009; Mennigen et al., 2009; Hormannsperger et al., 2010; Uronis et al., 2011; Isidro et al., 2017; Liu et al., 2019). VSL#3 was effective in preventing pouch colitis and inducing remission of ulcerative colitis in clinical trials (Gionchetti et al., 2003). A double-blind, randomized, placebo-controlled study showed that VSL # 3 could be used as adjunctive therapy for IBD and can be used in combination with standard 5-ASA or immunosuppressant therapy for the remission of relapsing mild-to-moderate ulcerative colitis (Tursi et al., 2010). In a meta-analysis of 23 randomized controlled trials, Shen et al. reported that VSL#3 significantly increased the remission rates of active UC. [P¼0.01, risk ratio (RR)¼1.51], and also considerably reduced the clinical recurrence rate of pouch colitis (P, 0.00001, RR¼0.18), without additional adverse events (Shen et al., 2014). Although VSL#3 reduced the levels of mucosal inflammation in patients with CD, there was no significant difference in the rate of endoscopic recurrence between patients who received VSL#3 and placebo. Therefore, whether VSL#3 can prevent the recurrence of Crohn’s disease remains to be validated (Fedorak et al., 2015).

Persborn et al. applied Ecologic^®^825 to UC patients with severe pouchitis founded that it normalized the permeation of *E. coli* K12, which was associated with active pouchitis, improved mucosal permeability, but had no effect on mucosal pouch microbiota composition (Persborn et al., 2013). Furrie et al. found that short-term synbiotic therapy (*Bifidobacterium longum*/Synergy 1) alleviated active UC by reducing inflammation and promoting epithelial tissue regeneration (Furrie, 2005). Steed et al. further confirmed that synbiotic therapy (*Bifidobacterium longum*/Synergy 1) effectively alleviated active Crohn’s disease (Steed et al., 2010). Fujimori et al. also found a combination of probiotics and prebiotics (*Bifidobacterium breve, Lactobacillus casei, Bifidobacterium Longum* and Psyllium) was effective against active Crohn’s disease without causing adverse events. Specifically, complete remission was observed in six patients, partial remission was observed in one patient, whereas three patients were non-responsive (Fujimori et al., 2007). In their clinical trial, Krag et al. found that Profermin^®^ (*Lactobacillus plantarum*299V, fermented oats, barley malt and lecithin) was fairly tolerable and promoted remission of UC without causing serious adverse reactions. The estimated reduction mean score was 5.0 points (95% CI: 4.1-5.9, P < 0.001) (Krag, 2012). SER-287, composed of Firmicutes polyspores, is safe and well-tolerated. SER-287 induced a high remission of moderate UC after vancomycin treatment and promoted bacterial colonization of gut microbiota (Henn et al., 2021). Besides prolonged active IBD remission, microbial combination therapy can modulate inflammation and maintain remission in patients with asymptomatic or quiescent IBD. Bjarnason et al. found that a multi-strain probiotic Symprove decreased intestinal inflammation in patients with asymptomatic UC (Bjarnason et al., 2019). Yoshimatsu et al. used Bio-Three tablets containing *Streptococcus faecalis, Clostridium butyricum* and *Bacillus mesentericus* to maintain clinical remission in patients with quiescent UC (Yoshimatsu et al., 2015). Caviglia et al. found that FEEDColon^®^ (consists of *Bifidobacterium bifidum, Bifidobacterium lactis*, calcium butyrate and oligosaccharides) was an effective adjunctive therapy for prolonged UC remission. Further analysis revealed that the remission rate of 5-ASA + FEEDColon^®^ was greater than of 5-ASA alone (95% > 57%) (P = 0.009) (Caviglia et al., 2021).

There are also several bacterial combinations against IBD, such as bacterial ecosystem therapeutic-2 [MET-2] (ClinicalTrials.gov, 2019) and IDOFORM TRAVEL^®^ (ClinicalTrials.gov, 2020a), under development (**Table 1**).

**Table 1:** Ongoing clinical trials of artificial consortium transplantation products.

Name	Components	Indication	ClinicalTrials.gov Identifier	Ref.
MET-2	comprises 40 different strains of gut bacteria from a healthy donor	Mild to moderate ulcerative colitis	NCT03832400	(ClinicalTrials.gov, 2019)
IDOFORM^®^Travel	*Lactobacillus rhamnosus* (LGG), *Lactobacillus acidophilus* (LA-5), *Bifidobacterium* sp. (BB-12), *Lactobacillus bulgaricus* (LBY-27), and *Streptococcus thermophilus* (STY-31)	patients with ulcerative colitis undergoing anti-TNF treatment with insufficient clinical response	NCT04241029	(ClinicalTrials.gov, 2020a)
Synbiotic	three Bifidobacterium spp.(*Bifidobacterium longum* spp. *longum* R0175, *Bifidobacterium animalis* spp. *Lafti* B94, *Bifidobacterium bifidum* R0071) plus three dietary fibers	Post-op Crohn’s Disease	NCT04804046	(ClinicalTrials.gov, 2021)
Probiotic Mixture	contains 8 different strains of bacteria, the specific composition is unclear	Quiescent Inflammatory Bowel Disease	NCT03266484	(ClinicalTrials.gov, 2017)
Probiotic Formula	*Lactobacillus rhamnosus, Lactobacillus acidophilus, Lactobacillus reuteri, Lactobacillus paracasei, Lactobacillus casei, Lactobacillus gasseri, Lactobacillus plantarum, Bifidobacterium lactis, Bifidobacterium breve, Bifidobacterium bifidum, Bifidobacterium longum, Bifidobacterium infantis*	Ulcerative colitis	NCT04223479	(ClinicalTrials.gov, 2020b)
Peptidic+ Probiotic	Oligomeric oral nutritional supplement (Bi1 peptidic), *Bifidobacterium animalis subsp. lactis* BPL1, *Lactobacillus rhamnosus* BPL15, *Lactobacillus rhamnosus* CNCM i-4036 *Bifidobacterium longum* ES1	Crohn’s Disease	NCT04305535	(ClinicalTrials.gov, 2020c)

## 4 Selection and Combination

### 4.1 Selection

Therapies relying on bacterial combinations usually use beneficial bacteria that have a research basis to support their benefits, most of which are recognized probiotics, such as Lactobacillus and Bifidobacterium (Araya et al., 2002). The bacteria used in combination therapy should be culturable and adaptable to the unique gastrointestinal environment. The bacteria must also stably colonize the intestinal tract.

#### 4.1.1 Environmental Adaptability

To reach therapeutic levels, the bacteria used in microbiota transplantation must be resistant to gastric acidity and bile acid toxicity (Daliri and Lee, 2015). Low pH is a primary host defense against ingested microorganisms. Compared with Bifidobacteria, Lactobacillus is more resistant to low pH (Tripathi and Giri, 2014). Acid resistance is not only genus-specific but also species-specific. For example, *L.casei* and *L. acidophilus* are better resistant to the low pH than *L.delbruekiis* sp.*bulgaricus*. Interestingly, the different Bifidobacterium strains vary in their resistance to gastrointestinal tract acidity (Daliri and Lee, 2015), with *B. animalis* the most acid-resistant strain (Saarela et al., 2006).

Acid tolerance is also linked to bacterial genetics. For instance, the loss of the urec gene encoding a protein associated with acid resistance reduced the ecological adaptability of *L. ruteri* 100-23 (Krumbeck et al., 2016). Bacterial bile salt hydrolase (BSH) protects intestinal bacteria from bile salts by breaking down conjugated bile salts into conjugated bile acids. Several bacterial genera, including Lactobacillus (Corzo and Gilliland, 1999; Wang et al., 2012), Bifidobacterium (Kim et al., 2004), Enterococcus (De Filippo et al., 2010) and Clostridium spp (Coleman and Hudson, 1995), secrete BSH. Therapeutic strains must be safe for use, even in immunocompromised individuals. And to avoid elimination by the gut immune response, probiotic strains usually have mild (not pro-inflammatory) immunomodulatory effects (Daliri and Lee, 2015). The environmental adaptability of strains also includes sensitivity to oxygen and utilization of nutrients.

#### 4.1.2 Colonization Characteristic

Stable colonization is a vital beneficial bacterial trait. Probiotics must adhere to the human intestinal cells and intestinal mucins to colonize and proliferate in these areas and compete with potential pathogens that adhere to mucosal surfaces. Recent studies have shown that the pattern of establishment, colonization and persistence of bacteria in certain gut sites are species-and strain-specific and are related to the host factors, strains characteristics, microbial interactions, and the diet (Xiao et al., 2021).

Introducing strains early in life or after antibiotic treatment promotes colonization and persistence of the bacteria in the intestines (Denou et al., 2008; Xiao et al., 2021). From the ecological perspective and coevolution, it is possible to determine the colonization and persistence potential of a particular bacterial species in human intestines. For example, judging from the natural history, compared with Lactobacillus, Bifidobacteria can colonize the intestinal tract more easily (Xiao et al., 2020). *B. longum* is a perfect example of bacterial species with long-term gut colonization potential, and it is dominant in the gut throughout the human lifespan (Odamaki et al., 2018). Bacterial genes, such as luxS (Tannock et al., 2005; Christiaen et al., 2014), pili (Turroni et al., 2013), and BSH (Corzo and Gilliland, 1999), are essential in host-microbe interactions. For instance, the luxS gene in *B. breve* UCC2003 (Christiaen et al., 2014)and *L. reuteri* 100–23 (Tannock et al., 2005) participated in producing the interspecies signaling molecule autoinducer-2 (AI-2), which mediates colonization of the bacteria in the human gut. Pili is a strain-specific colonization factor in LGG (but not in LC705) support the intestinal colonization ability of bacteria was strain-specific (Kankainen et al., 2009). In addition to the bacterial characteristics that contribute to its colonization, specific strains display reciprocal colonization effect and may influence the colonization of other strains. The success of FMT in intestinal flora reconstruction after antibiotic treatment suggests that a flora with rich genetic diversity and metabolic interactions is more likely to thrive in the intestine (Suez et al., 2018). Moreover, cross-feeding of Bifidobacterium strains (*B. bifidum*PRL2010, *B. breve*12L, *B. adolescentis*22L, and *B. Infantis*ATCC15697) improved the persistence of each strain in the cecum (Turroni et al., 2016). Therefore, exploring the interaction behavior of probiotic strains can enhance the development of effective co-colonization combination strains. The core effect of dietary is by adding prebiotics to provide privileged nutrition pathway for intake of strains. Prebiotics including tryptophan, GOS and polysaccharide, which enhanced the colonization of specific bacterial strains. High levels of tryptophan increased the abundance of *L. reuteri* in the stomach and stool (Zelante et al., 2013), whereas high levels of GOS in the human intestine significantly enrich the abundance of Bifidobacterium in the gut in a dose-dependent manner (Krumbeck et al., 2015). Despite these findings, additional synergistic dietary and specific bacterial combinations that enhance the colonization of beneficial bacteria in the gut need to be identified.

### 4.2 Bacterial Combination

#### 4.2.1 Types of ACT Products

In this article, the current bacterial combinations for IBD were divided into four types (**Table 2**). The first type is a combination of probiotics combined with prebiotics, named synbiotic. Prebiotics are dietary fiber supplementations that stimulate the growth of specific, putatively beneficial bacteria already present in the gut. Prebiotics promote the metabolism and colonization of probiotics. The most commonly used prebiotics are fermentable carbohydrates such as inulin, oligosaccharides, galactose oligosaccharides and resistant starch. In addition, polyphenols and polyunsaturated fatty acids, which act on gut microbes, are also classified as prebiotics. Example of this type of combination include Profermin^®^ (Krag, 2012), FEEDColon^®^ (Caviglia et al., 2021) and YBF (ClinicalTrials.gov, 2010).

**Table 2:** Four categories of artificial consortium transplantation products.

Type	Name	Components	Producer	Ref
**Synbiotic**	Lactobacillus sp. +prebiotic	grape pomace extract, *L. rhamnosus* (IDIBNA02), *L. paracasei* (ID13239), *L. acidophilus* (ID11692)	Gina Cecilia Pistol et al.	(Pistol et al., 2019)
	*Bifidobacterium longum*/Synergy 1	Fructo-oligosaccharide/inulin mix, *B. longum*	H. Steed et al	(Steed et al., 2010)
	Profermin^®^	Fermented oats, barley malt, lecithin, *L.plantarum* 299v	Nordisk Rebalance	(Krag, 2012)
	Symprove™	Barley extract, *L.rhamnosus* NCIMB 30174, *L. plantarum* NCIMB 30173, *L. acidophilus* NCIMB 30175, *Enterococcus faecium* NCIMB 30176	Symprove Ltd	(Bjarnason et al., 2019)
	Bio-Three tablets	Potato starch, lactose, *Streptococcus faecalis* T-110, *Clostridium butyricum* TO-A, *Bacillus mesentericus* TO-A	Toa Pharmaceutical Co.	(Yoshimatsu et al., 2015)
	FEEDColon^®^	Calcium butyrate, fructo-oligosaccharides, *B. bifidum*, *B. lactis*	Princeps	(Caviglia et al., 2021)
	YBF	Yogurt, soluble fiber, Bifidobacteria	Instituto Lala	(ClinicalTrials.gov, 2010)
**Mutualbiotic**	VSL#3	*L. plantarum, L. paracasei, L. delbrueckii subsp. bulgaricus, L. acidophilus, B. longum, B. breve, B. infantis*, *Streptococcus thermophilus*	VSL#3, Pharma	(Reiff et al., 2009)
	Visbiome^®^	*L. plantarum* DSM 24730, *L. paracasei* DSM 24733, *L. delbrueckii subsp. bulgaricus* DSM 24734, *L. acidophilus* DSM 24735, *B. longum* DSM 24736, *B. breve* DSM 24732, *B. infantis* DSM 24737, *Streptococcus thermophilus* DSM 24731	A blend produced under Prof. De Simone’s control	(Rossi et al., 2018)
	Five strains probiotics	*L. casei, B. breve, B. animalis subsp. Lactis*, *Streptococcus thermophilus, Bacillus subtilis*	Michele Biagioli et al.	(Biagioli et al., 2020)
	Ultrabiotique^®^	*L. acidophilus, L. plantarum, B. lactis, B. breve*	Laboratoire Nutrisante	(Toumi et al., 2013)
	Citogenex	*L. casei, B. animalis subspecies lactis*	G Traina et al.	(Traina G, 2016)
	PM-2	*L. acidophilus, L. paracasei, L. rhamnosus, B. lactis*	Marielle Quaresma et al.	(Quaresma et al., 2020)
	ID-JPL934	*L. johnsonii*IDCC9203, *L. plantarum*IDCC3501, *B. animalis subspecies lactis*IDCC4301	In-Gyu Je et al.	(Je et al., 2018)
	I3.1 probiotic	*L. plantarum* (CECT7484, CECT7485), *Pediococcus acidilactici* (CECT7483)	AB-Biotics S.A	(Lorén et al., 2017)
	GI7	*L. acidophilus* LA1 (KCTC 11906BP), *L. plantarum* LP3 (KCTC 10782BP), *L. rhamnosus* LR5 (KCTC 12202BP), *L. lactis* SL6 (KCTC 11865BP), *B. bifidum* BF3 (KCTC 12199BP), *B. breve*BR3 (KCTC 12201BP), *Streptococcus thermophilus* ST3 (KCTC 11870BP).	M.S. Kim et al.	(Kim et al., 2017)
	Ecologic^®^825	*L. acidophilus, L. casei, L. paracasei, L. plantarum*, *L. salivarius, B. bifidum, B. lactis, Lactococcus lactis.*	Winclove Probiotics BV	(Persborn et al., 2013)
	IDOFORM^®^Travel	*L. rhamnosus* (LGG), *L. acidophilus* (LA-5), *L. bulgaricus* (BY-27), *Bifidobacterium* sp. (BB-12), *Streptococcus thermophilus* (STY-31)	Pfizer	(ClinicalTrials.gov, 2020a)
	SYNBIO ^®^	*L. rhamnosus* IMC 501^®^, *L. paracasei* IMC 502^®^	Synbiotec S.r.l.	(Coman et al., 2020)
	LAB mixture	*L. plantarum* CRL 2130, *Streptococcus thermophilus* (CRL 807, CRL 808)	Romina Levit et al.	(Levit et al., 2019)
	Bifico	Bifidobacterium, Lactobacillus, Enterococcus	Shanghai Sine Pharmaceutical	(Zhang et al., 2018)
	Bio 25	*L. rhamnosus* LR5, *L. casei* LC5, *L. paracasei* LPC5, *L. plantarum* LP3, *L. acidophilus* LA1, *L. bulgaricus* LG1, *B. bifidum* BF3, *B. longum* BG7, *B. breve* BR3, *B. infantis* BT1, *Streptococcus thermophilus* ST3, *Lactococcus lactis* SL6	Supherb Ltd	(ClinicalTrials.gov, 2013)
**Diverbiotic**	BCT	Lactobacillus, Eubacterium, Pediococcus, Veillonella, Streptococcus, Staphylococcus, Bifidobacterium, Bacteroide, Escherichia, Fusobacterium	Ming Li et al.	(Li et al., 2015)
	GUT-103	*Megamonas funiformis* DSM19343, *Megamonas hypermegale* DSM1672, *Acidaminococcus intestini* DSM21505, *Bacteroides massiliensis* DSM17679, *Bacteroides stercoris* ATCC43183/DSM19555, *Barnesiella intestinihominis* DSM21032, *Faecalibacterium prausnitzii* DSM17677, *Subdoligranulum variabile* DSM15176, *Anaerostipes caccae* DSM14662, *Anaerostipes hadrus* DSM3319/ATCC 29173, *Clostridium symbiosum* ATCC14940, *Akkermansia muciniphila* ATCC BAA-835, *Clostridium scindens* ATCC35704, *Clostridium bolteae* ATCC BAA-613, *Blautia producta* DSM2950, *Blautia hydrogenotrophia* DSM10507, *Marvinbryantia formatexigens* DSM14469	Daniel van der Lelie et al.	(van der Lelie et al., 2021)
	GUT-108	*Bacteroides xylanisolvens* GGCC_0124, *Clostridium butyricum*GGCC_0151, *Clostridium scindens* GGCC_0168, *Intestinimonas butyriciproducens* GGCC_0179, Extibacter sp.GGCC_0201, *Eubacterium callanderi* GGCC_0197, *Akkermansia* sp. GGCC_0220, *Clostridium symbiosum* GGCC_0272, *Bacteroides uniformis*GGCC_0301, *Bitterella massiliensis* GGCC_0305, *Barnesiella* sp. GGCC_0306	Daniel van der Lelie et al.	(van der Lelie et al., 2021)
	SER-287	Spores of Firmicutes	Matthew R. Henn et al.	(Henn et al., 2021)
	MET-2	40 different strains of gut bacteria from a healthy donor	NuBiyota	(ClinicalTrials.gov, 2019)
**Ejusbiotic**	Butyrate-producing bacteria	*Butyricicoccus pullicaecorum* 25-3T (LMG 24109 T), *Butyricicoccus pullicaecorum* 1.20, *Faecalibacterium prausnitzii* (DSM 17677), *Roseburia hominis* (DSM 16839), *Roseburia inulinivorans* (DSM 16841), *Anaerostipes caccae* (DSM 14662) and *Eubacterium hallii* (DSM 3353)	Annelies Geirnaert et al.	(Geirnaert et al., 2017)

L., Lactobacillus; B., Bifidobacteriu.

The second type of ACT products comprises beneficial bacteria of different phyla or genera paired based on their social interactions, is a mutual effect combination (mutualbiotic). The most common match includes a combination of Bifidobacteria or Lactobacillus and other strains. The strains establish a symbiotic interaction that promotes reciprocal colonization in the harsh and dynamic human gut environment, including ID-JPL934 (Je et al., 2018), GI7 (Kim et al., 2017), VSL#3 (Tursi et al., 2010) and IDOFORM TRAVEL^®^ (ClinicalTrials.gov, 2020a).

The third type of diverse bacterial combination consists of strains from multiple phyla, named diversified combination(diverbiotic). These strains are isolated from feces then combined in an optimum manner to form a diverse community. This type of consortia includes BCT (Li et al., 2016), GUT-103, GUT-108 (van der Lelie et al., 2021) and MET-2 (ClinicalTrials.gov, 2019).

The fourth type of bacterial consortia includes a combination of bacteria grouped together according to metabolic characteristics, named ejusdem combination (ejusbiotic), comprises bacteria that produce the same metabolic products beneficial for IBD patients. An example is the butyrate-producing bacteria (Geirnaert et al., 2017).

#### 4.2.2 The Principles of the ACT Products

ACT shall be developed in accordance with the principles of complementarity, reciprocity, specificity and stability (CRSS).

The function of each strain is limited, and the strains complement the effects of each strain. For example, the butyrate-producing bacteria consortium competed with resident microbiota for substrates (e.g., acetate) and, thus, promoted the production of butyrate (Geirnaert et al., 2017).

**Figure 2:**
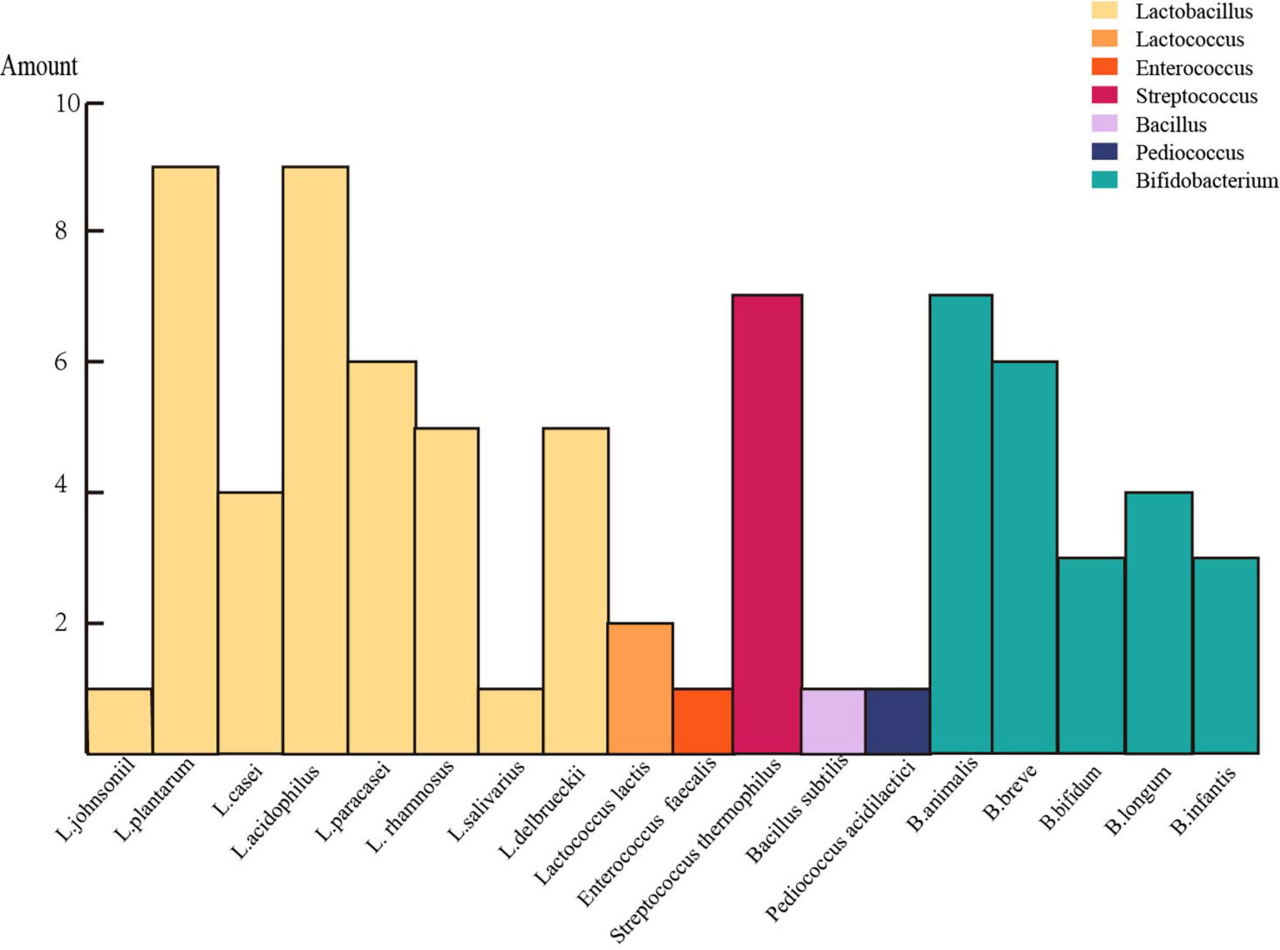
A chart listed the number of occurrences of each strain in these mutualbiotics (one color represents a genus).

The gut microbiome must establish a reciprocal interaction to remain stable and functional in the dynamic gut environment. Given that exogenous acetate is critical in maintaining butyrate production, the cross-feed between the acetic acid-producing bacteria and butyrate-producing bacteria enhances the continued functioning of butyrate-producing bacteria (Duncan et al., 2004). The cross-feeding observed among the intestinal microbiota suggests the inter-dependence of the strains. Lactobacillus and Bifidobacterium, the most commonly used probiotics, are the most common artificial consortium bacteria (**Table 3**; **Figure 2**. each corresponding). Most Bifidobacteria display reciprocal carbohydrate metabolism capability *in vitro (* Riviere et al., 2018) and persistence in the gut *in vivo (* Turroni et al., 2016). In addition to their symbiosis association within the genus, Bifidobacterium promotes the activities of other gut bacteria (e.g., Bacteroidetes), including carbohydrate metabolism, thus, enhancing the environmental adaptability of these bacteria (Sonnenburg et al., 2006; Turroni et al., 2016).

**Table 3:** A list of each product components of mutulbiotics applied in IBD.

Phylum	Firmicutes													Actinobacteria				
Genus	Lactobacillus								Lactococcus	Entero-coccus	Streptococcus	Bacillus	Pediococcus	Bifidobacterium				
Species/Name	johnsonii	plantarum	casei	acidophilus	paracasei	rhamnosus	salivarius	delbrueckii	lactis	faecalis	thermophilus	subtilis	acidilactici	animalis	breve	bifidum	longum	infantis
VSL#3		O		O	O			O			O				O		O	O
Visbiome^®^		DSM 24730		DSM 24735	DSM 24733			Bulgaricus DSM 24734			DSM 24731				DSM 24732		DSM 24736	DSM 24737
Five strains probiotics			O(30%)								O(30%)	O(10%)		lactis15%	O(15%)			
Ultrabiotique^®^		O		O										lactis	O			
Citogenex			O											lactis				
PM-2				O	O	O								lactis				
ID-JPL934 (1:1:1)	DCC9203	DCC3501												lactisDCC4301				
I3.1probiotic formula		CECT7484+ CECT7485											CECT7483					
GI7		LP3(KCTC 10782BP)		LA1(KCTC 11906BP)		LR5 (KCTC 12202BP)		LactisSL6 (KCTC 11865BP)			ST3(KCTC 11870BP)				BR3(KCTC12201BP)	BF3 (KCTC12199BP)		
Ecologic^®^825		W62	W56	W22	W20		W24		W19					lactisW51+W52		W23		
IDOFORM^®^ Travel				LA-5		O		bulgaricusLBY-27			STY-31			BB-12				
SYNBIO^®^					IMC50^®^	IMC501^®^												
LAB mixture		CRL 2130									CRL808+ CRL 807							
Bifico				O						O							O	
Bio 25		LP3	LC5	LA1	LPC5	LR5		LG1	SL6		ST3				BR3	BF3	BG7	BT1

“O” indicates the existence of the strain in this product, but the specific strain is not clear.

The strain-specific and disease-specific effects of bacteria on disease have been extensively reported. In addition to the inter-species differences, the diversity within species should be considered. For example, in describing two different *F. prausnitzii* phylogroups (Lopez-Siles et al., 2012), Lopez-Siles et al. found that whereas the abundance of phylogroup I was significantly low in the gut of CD, UC, and colorectal cancer patients, depletion of phylogroup II was explicitly related to CD (Lopez-Siles et al., 2016). A decrease in Lactobacillus is predominant in active ulcerative colitis, whereas a similar phenomenon is observed for Bifidobacteria in Crohn’s disease.

Conditions may also influence the effect of the final ACT products, such as production method or strain ratio. Even if the composition of strains in the artificial consortium products is similar, their effect may differ. For example, VSL#3 and Visbiome^®^ are two products composed of the same bacteria species. However, Visbiome^®^ activated Treg cells (CD4+FoxP3+) and T lymphocytes to produce anti-inflammatory cytokines IL-10 and short-chain fatty acids more effectively than VSL#3, which may be due to different production methods or composition ratios of individual bacterial species (Biagioli et al., 2019). Therefore, more stable production standards should be maintained.

## 5 Perspectives of Engineered Consortium Transplantation

Naturally isolated strains aside, genetically modified bacteria can be used for microbiota transplantation (Gao et al., 2022). The engineered strains perform different functions from the original strain or possess modified metabolic characteristics. For example, Puurunen et al. inserted genes encoding phenylalanine ammonia-lyase and L-amino acid deaminase into the *E. coli Nissle* 1917 genome, generating the modified SYNB1618 strain. The engineered strain could degrade phenylalanine in the gastrointestinal tract (Puurunen et al., 2021). A butyrate-producing bacteria, recombinant *B. subtilis* BsS-RS06550 with high butyric acid production was constructed using synthetic biological strategies could effectively regulate body metabolism and intestinal flora disruption (Bai et al., 2020; Wang et al., 2022).The modified strains can increase the variety and number of available strains to the bacterial combination. So far, the engineered strain alone against certain diseases, such as phenylketonuria (Puurunen et al., 2021), liver cirrhosis (https://clinicaltrials.gov/ct2/show/NCT03447730?term=NCT03447730&draw=2&rank=1), has been assessed.

*Lactococcus lactis* (LL-Thy12), in which the thymidylate synthase gene was replaced with a synthetic sequence encoding mature human interleukin-10 (Braat et al., 2006), was found to alleviate Crohn’s disease. Yeast expressing human P2Y2 purinergic receptor and ATP-degrading enzyme, creating self-regulating yeast probiotics system capable of sensing pro-inflammatory molecules inhibits intestinal inflammation in IBD mice (Scott et al., 2021). However, clinical trials on the efficacy of engineered strains against IBD are limited, and even few engineered strains have been transplanted together.

Bacterial combinations promote metabolic characteristics and colonization and increase species diversity. Therefore, the efficacy of the combination of engineered bacteria is a promising research direction for microbiota transplantation.

## 6 Discussion

Autochthonous strains are more likely to exert beneficial effects, while allochthonous strains may stimulate the immune system to some extent (Tannock et al., 2000; Yamashita et al., 2020). The sources of the strains are various, such as fermented food and feces. While from the co-evolution perspective, it is better to select strains isolated from human feces for transplantation into the human intestine. Different species display distinctly varied gut fitness. For instance, autochthonous lactobacilli showed better gut colonization ability than the allochthonous (Duar et al., 2017). Microbiota transplantation research is gravitating toward using specific FMT or engineered fecal microbiota, which generates a superior effect to the natural fecal microbiota.

The efficacy of strain combinations can be assessed based on the degree and duration of *in vivo* colonization. Most of the data on intestinal colonization of bacteria have been derived from animal models. Given the differences between animal and human systems, more clinical trials are needed to validate the effectiveness of combined microbiota transplantation. Moreover, the functional characterization of most symbiotic strains in the gut is still in infancy, and more research is needed to identify new strains with high potential for health benefits. Identification of novel health-associated gut bacteria allows better insight into the functionality of the different species and strains (Cuffaro et al., 2021). The findings extend the number of potential candidates for personalized probiotics, taking individual host variations and specific responses into account.

The diversity of gut microbiota is critical for maintaining resilience, and therefore, the transplantation of microbiota combinations is a potentially effective alternative for IBD treatment. Previous articles on microbiota transplantation were mainly limited to FMT, and most of them focused on the application of FMT in IBD, or emphasized the importance of the gut microbes in the pathogenesis and treatment of IBD (Zuo and Ng, 2018; Ooijevaar et al., 2019; Tan et al., 2020; Lee and Chang, 2021; Underhill and Braun, 2022; Halaweish et al., 2022). We mainly analyzed different microbiome-based interventions currently applied in IBD clinical trials, including FMT, WMT (a method that removes adverse factors in natural FMT by special washing manner), as well as ACT, which combines different and limited microorganisms, and analyzed the possible combination principles of ACT. In particular, engineered single bacteria have been used against IBD in recent years (Braat et al., 2006; Scott et al., 2021), we imaged that the artificial consortium combined with engineered bacteria is expected to bring revolutionary mutations to microbiota transplantation.

Given the enormous prospective of microbiota transplantation, the review of different combinations and principles will help to provide a theoretical basis for the generation of more artificial consortium transplantation in the future. The application of microbiota combination in different disease states is differ, even distinct in different individuals. The artificial consortium should not just be a simple combination of strains. However, it should be oriented by engineering ideas and form a systematic whole with the combined characteristics of strains, classification of recipient microbes, disease stages and other factors. Such a consortium could be the next generation of microbiota transplants. Finding rules or principles on the basis of existing research is helpful in exploring the optimal solution of the microbiota approach applied to IBD patients, that is, fewer adverse effects and better clinical outcomes. Recently, Gianluca Ianiro published a comment on the treatment of recurrent *Clostridioides difficile* Infection by SER-109, an artificial microbiome consortium Product, which was similar to our idea in this review, that ACT, to a certain extent, overcome issues related to donor safety and maintenance associated with classical FMT. However, still needed more studies to compare synthetic microbial complexes with standard FMT. If these are better or equivalent to classic FMT, then this will herald the era of FMT2.0 (Feuerstadt et al., 2022; Ianiro, 2022).

The limitations of this review are that neither the fungal microbiome is taken into account nor receptor factors are combined. In most clinical trials, only limited information about the receptors has been mentioned. Generally, there is only a classification of IBD severity. However, the classification of the intestinal microbiota of the recipients is always absent and the age and gender information is also ominous, which is not conducive to our more comprehensive analysis. Therefore, we appeal to record more complete and comprehensive information in clinical trials which could provide a foundation for more comprehensive analysis of microbiota transplantation in the future.

## 7 Conclusion

**Figure 3:**
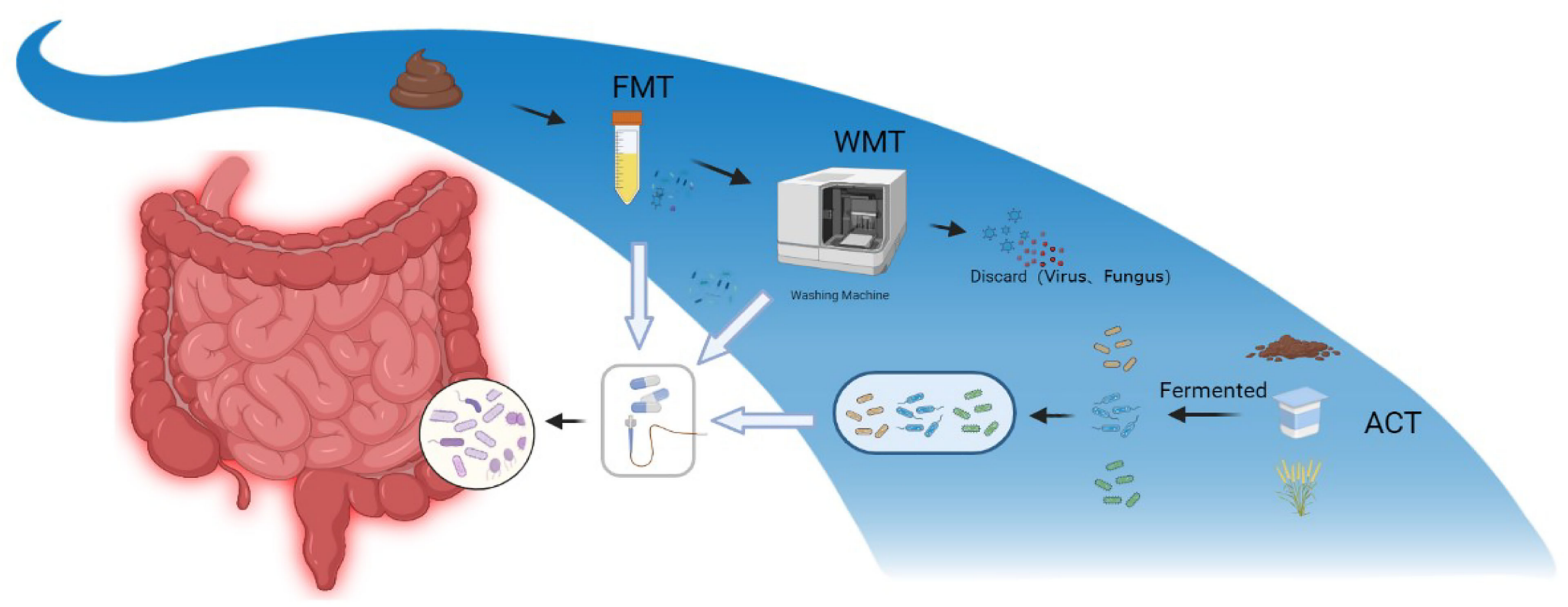
Diagram of three different modes of microbiota transplantation. FMT, fecal microbiota transplantation; WMT, washed microbiota transplantation; ACT, artificial consortium transplantation.

In this review, we summarized clinical studies on various microbiota combinations applied to IBD (**Figure 3**) and emphasized the application of artificial microbiota combination transplantation against IBD. The advantages of the bacterial combination were discussed, the types and the possible principles of ACT products were summarized, while the prospect of microbiota transplantation was discussed. The combination of microbiota needs to take the complementary relevance between strains, individual strains’ specificity and stable conditions into account. Future research should identify combinations of strains that display metabolic interactions based on ecological knowledge, bacterial genomic data and *in vitro* experimental results, and then validate them *in vivo*, ultimately contributing to mutually beneficial human implantation. The transplantation of microbiota combinations is a potentially safe and effective alternative for IBD treatment. Compared with classical FMT, ACT reduces safety concerns and diversified the options available for different disease states to some extent. Furthermore, the artificial consortium combined with engineered bacteria is expected to bring revolutionary mutations to the microbiota transplantation, which has a bright foreground against IBD. These will probably herald the arrival of the new era of microbial therapy!

## Author Contributions

XC, GK, XW, and JZ contributed to the conception of this review. XW and JZ wrote the first draft of the manuscript. YF, ZF, YY, and LL is responsible for literature retrieval. XC, GK, XW, JZ, YF, ZF, YY, and LL wrote sections of the manuscript. XC and GK supervise the project administration. All authors contributed to the article and approved the submitted version.

## Funding

The present study was supported by grants from the National Key Research and Development Project (GrantNo.2019YFA0905600); the Tianjin Health Science and Technology Research Project (GrantNo.TJWJ2021MS005); the Major State Basic Research Development Program of the Natural Science Foundation of Shandong Province in China (GrantNo.ZR2020ZD11), We thank Shaoxing “Ming Shi Zhi Xiang” Meritocrat Project and Program of Introducing Talents of Discipline to University Ministry of Education, China-111 Project (GrantNo.BP0618007) for its support.

## Conflict of Interest

The authors declare that the research was conducted in the absence of any commercial or financial relationships that could be construed as a potential conflict of interest.

## Publisher’s Note

All claims expressed in this article are solely those of the authors and do not necessarily represent those of their affiliated organizations, or those of the publisher, the editors and the reviewers. Any product that may be evaluated in this article, or claim that may be made by its manufacturer, is not guaranteed or endorsed by the publisher.
